# Independent Multi-states of Photo-responsive Polymer/Quantum Dot Nanocomposite Induced via Different Wavelengths of Light

**DOI:** 10.1038/s41598-019-48834-6

**Published:** 2019-08-28

**Authors:** Jiyeon Lee, Wonsik Lee, Dongjun Kim, Myungjun Kim, Jiwon Kim

**Affiliations:** 1 School of Integrate Technology, College of Engineering, Yonsei University, 85 Songdogwahak-ro, Yeonsu-gu, Incheon, 21983 Republic of Korea; 2 Integrated Science and Engineering Division, Underwood International College, Yonsei University, 85 Songdogwahak-ro, Yeonsu-gu, Incheon, 21983 Republic of Korea

**Keywords:** Quantum dots, Organic-inorganic nanostructures, Actuators, Polymers

## Abstract

Stimuli-responsive systems are attractive since their properties can be controlled by external stimuli and/or surrounding environment. Recently, more than one stimulus is utilized in order to enhance the performance of systems, or to bypass undesired effects. However, most of previous research on multi-stimuli has been focused on enhancing or inducing changes in one type of response. Herein, we developed a nanocomposite material with independent multi-states composed of photo-responsive polymer and quantum dots (QDs), in which its properties can independently be controlled by different wavelengths of light. More specifically, azobenzene-incorporated poly(dimethylsiloxane) (AzoPDMS) triggers photobending (PB) by 365 nm light and uniformly dispersed methylammonium lead bromide perovskite (MAPbBr_3_) QDs show photoluminescence (PL) by light below 500 nm. The PB and PL could be simultaneously and independently controlled by the wavelength of applied light creating multi-states. Our approach is novel in that it creates multiple independent states which can further be used to transfer information such as logic gates (00_(2)_, 01_(2)_, 10_(2)_, 11_(2)_) and possibly widen its application to flexible and transparent opto-electric devices.

## Introduction

Stimuli-responsive materials show changes in their shape^1,2^ or color^3–5^, generate heat^6–8^, associate/dissociate^9,10^, or decompose into compartments^11–13^ via various mechanisms. These materials can further be optimized to reversibly switch between ON and OFF states. Furthermore, more than one stimulus has been applied to enhance the performance of system by combining changes in the property of materials or to bypass undesired effect/environment by sequential reactions. When two or more stimuli are applied to the material, characteristic changes by each stimulus can be combined to enhance changes in the property (e.g. more precise control or increase in intensity). For instance, Wang *et al*. induced multi-directional bending of nanocomposite via humidity, heat, chemical gas, and light^14^. Das *et al*. expanded the complexity of nanoparticle self-assembly by stimulating with both light and magnetic field^15^. Jeon *et al*. combined pH and electrical field to ensure color diversity of gold nanostructures by inducing plasmonic resonance^16^. On the other hand, multi-stimuli can also be applied to detour undesired reaction or minimize damages from surrounding environment being hyperthermia as a representative example. Light^17–20^ and alternating magnetic field^21,22^ were used to locally induce hyperthermia in order to avoid applying heat to whole system (e.g. cells).

Among various stimuli, light is one of the most attractive stimuli since it can spatio-temporally and non-invasively control the property of materials by varying its wavelength or intensity. Moreover, if different wavelengths of light independently result in more than one response via different mechanisms, it can be considered as multi-stimuli. Property changes induced by light include photobending (PB)^19^, photoluminescence (PL)^23^, photodeformation (PD)^17^, and photoelectricity (PE)^24,25^ rooting from photoisomerization or photoexcitation as a key mechanism^26^.

More specifically, PB is commonly caused by isomerization of aligned photo-responsive molecules (e.g. azobenzene), or difference in volume change of each layer – bilayer film composed of photo-responsive and supporting layers – induced by light^1,27–31^. PL is often observed with QDs, and organometallic halide perovskite quantum dots (OHP QDs) are actively studied owing to its outstanding optical properties (e.g. narrow spectral width, high color purity, high quantum yield, and tunable emission)^32^. However, since OHP QDs often suffer from poor stability, many studies have been reported to stabilize OHP QDs by passivating them with polymer^33–36^.

Herein, we developed multi-switchable nanocomposite film with two independent properties, which could be controlled by different wavelengths of light. One of the two properties is PB, which was implemented by photo-switchable molecule, azobenzene, incorporated polymer (poly(dimethylsiloxane); PDMS); the photo-responsive polymer changed its structure by light of specific wavelength. The other property is PL which was realized by OHP QDs; more specifically, methylammonium lead bromide (CH_3_NH_3_PbBr_3_; MAPbBr_3_) QD was used which was more stable compared to other halide (e.g. Cl, I) OHP QDs.

By combining abovementioned materials – azobenzene incorporated poly(dimethylsiloxane) (AzoPDMS) and MAPbBr_3_ QDs, we have synthesized multi-switchable nanocomposite film which showed PB and PL independently via different wavelengths of light. The fabricated MAPbBr_3_ QD/AzoPDMS composite film exhibited four states: (i) luminescence OFF & stretched (initial state), (ii) luminescence OFF & bent (λ > 500 nm), (iii) luminescence ON & stretched (λ = 365 nm), and (iv) luminescence ON & bent (λ > 365 nm). The nanocomposite film was capable of performing bending and luminescence simultaneously without any additional physical contact. We believe it can be applied to not only opto-electric devices such as bioelectronics or electrical actuators, but also signal processing devices such as logic gates. Furthermore, we believe the independently created multi-states could also mimic more complex (biological) reactions or natural phenomena.

## Results and Discussion

**Figure 1:**
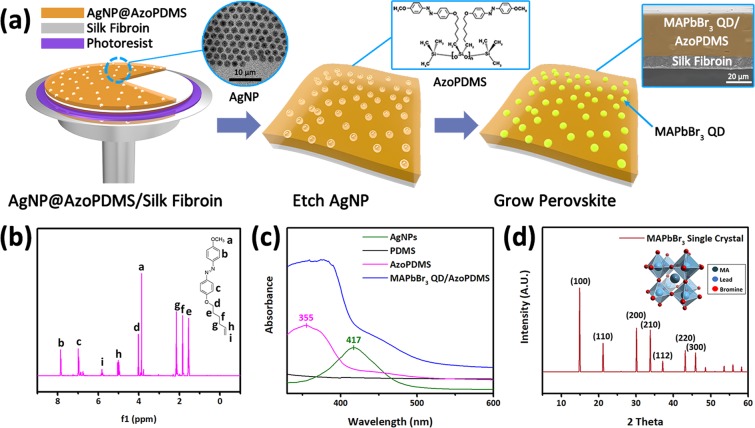
Synthesis and characterization of MAPbBr_3_ QD/AzoPDMS composite film. (**a**) Fabrication scheme of MAPbBr_3_ QD/AzoPDMS composite film. Transmission electron microscopy (TEM) image of AgNPs, molecular structure of AzoPDMS, and scanning electron microscopy (SEM) cross section image of MAPbBr_3_ QD/AzoPDMS composite film are included as insets of the scheme. SEM image showed bilayer structure of MAPbBr_3_ QD/AzoPDMS and silk fibroin. (**b**) Nuclear magnetic resonance (NMR) spectrum of the synthesized azobenzene molecule. (**c**) Ultraviolet-visible (UV-Vis) absorption spectra of AgNPs, PDMS, AzoPDMS, and MAPbBr_3_ QD/AzoPDMS composite film. (**d**) X-ray diffraction (XRD) analysis of MAPbBr_3_ single crystal.

The fabrication process of MAPbBr_3_ QD/AzoPDMS and silk fibroin bilayer film is illustrated in Fig. 1(a). First of all, 1-(4-(hex-5-enyloxy)phenyl)-2-phenyldiazene, an azobenzene derivative, was incorporated within the poly(dimethylsiloxane) (PDMS) backbone chain during the polymerization process where the molecular structure of AzoPDMS is shown in Fig. 1(a). Silver nanoparticles (AgNPs) were then blended with AzoPDMS prepolymer in order to synthesize porous AzoPDMS template followed by etching process. More detailed process can be found in Experimental section. To note, AgNPs were used instead of gold nanoparticles (AuNPs) as in our previous study^35^, since AzoPDMS was fragile in gold etchant which is a strong acid (i.e. aqua regia). The diameter of AgNPs were 5.3 ± 0.6 nm imaged by transmission electron microscopy (TEM) as shown in the inset of Fig. 1(a). AgNPs were etched away with 3 M nitric acid, and MAPbBr_3_ QDs were grown in spherical voids of AgNP size. As a result, 32.4 μm thick MAPbBr_3_ QD/AzoPDMS layer was fabricated on the 10 μm thick silk fibroin layer as shown in the cross section of bilayer (inset of Fig. 1(a)). Nuclear magnetic resonance (NMR) spectrum of the synthesized azobenzene molecule was also obtained to confirm the molecular structure, and each peak is assigned as shown in the Fig. 1(b). Ultraviolet-visible (UV-Vis) absorption spectra of AgNPs, PDMS, AzoPDMS, and MAPbBr_3_ QD/AzoPDMS composite are shown in Fig. 1(c) for a comparison. Absorption spectrum of AzoPDMS showed peak at 355 nm which was nearly the same as an azobenzene precursor, 1-(4-(hex-5-enyloxy) phenyl)-2-phenyldiazene, which also indicates it has azobenzene moiety within the structure. AzoPDMS absorbed light of 340–370 nm and isomerized from *trans*-form to *cis*-form, therefore we used 365 nm band pass filter (transmission from 360 to 372 nm, more detail in Experimental Section) to selectively apply the wavelength of light for PB. The absorption peak of AgNPs disappeared in the spectra of MAPbBr_3_ QD/AzoPDMS composite film since they were etched away during the fabrication process. The synthesized MAPbBr_3_ crystal which was grown inside the porous AzoPDMS showed X-ray diffraction (XRD) peaks at 14.98°, 21.22°, 30.16°, 33.82°, 37.3°, 43.28°, and 45.92°, which are indexed to the (100), (110), (200), (210), (112), (220), and (300) planes, respectively (Fig. 1(d))^35^.

**Figure 2:**
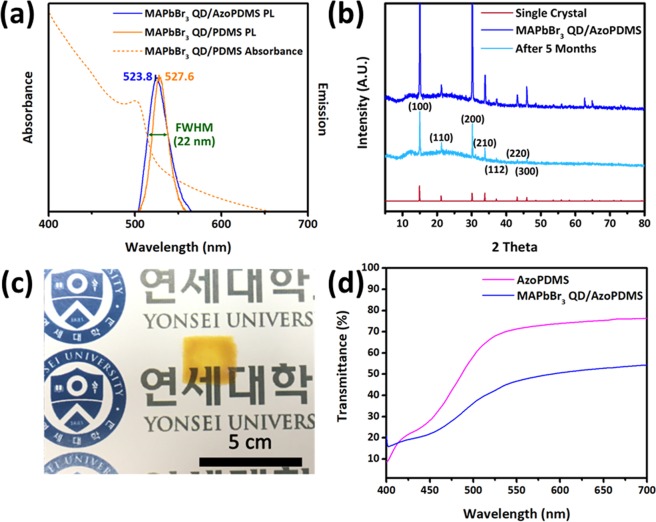
Photoluminescence (PL), stability, and transmittance of MAPbBr_3_ QD/AzoPDMS composite film. (**a**) PL spectra of MAPbBr_3_ QD/PDMS composite and MAPbBr_3_ QD/AzoPDMS composite. MAPbBr_3_ QD/AzoPDMS composite film exhibited λ_max_ at 523.8 nm by absorbing light with a wavelength below 500 nm. (**b**) XRD analysis of MAPbBr_3_ QD/AzoPDMS composite film showed that the film is stable for at least a few months. (**c**) A photograph of transparent MAPbBr_3_ QD/AzoPDMS composite film. (**d**) Transmittance spectra of MAPbBr_3_ QD/PDMS composite and MAPbBr_3_ QD/AzoPDMS composite which showed transmittance of ~76% and ~53% at 700 nm, respectively.

MAPbBr_3_ QDs embedded in AzoPDMS emitted light of 523 nm as shown above, which was consistent with our previous study (Fig. 2(a))^35^. Moreover, MAPbBr_3_ QD/AzoPDMS prepared in this work showed narrow full width at half maximum (FWHM) of about 22 nm which indicates that the size of QDs grown inside the pores is uniform. 460 nm light was irradiated to MAPbBr_3_ QD/AzoPDMS composite film for PL measurement, which is included in the range of absorbance measurement (below the 500 nm) as shown in Fig. 2(a). For MAPbBr_3_ QDs grown inside PDMS, PL spectrum with a peak at 527 nm was observed under 460 nm light irradiation which is similar to previously reported works. The XRD peaks of MAPbBr_3_ QDs were also observed at 14.98°, 21.22°, 30.16°, 33.82°, 37.3°, 43.28°, and 45.92°, respectively, which was the same with MAPbBr_3_ single crystal (Fig. 2(b))^35^. The MAPbBr_3_ QDs embedded in AzoPDMS matrix were protected from external environment maintaining stability for at least 5 months as shown by the same XRD graph pattern with newly prepared sample in Fig. 2(b). Prepared MAPbBr_3_ QD/AzoPDMS composite film was transparent as shown in Fig. 2(c) with transmittance of ~53% while that of AzoPDMS was ~76% at 700 nm (Fig. 2(d)).

**Figure 3:**
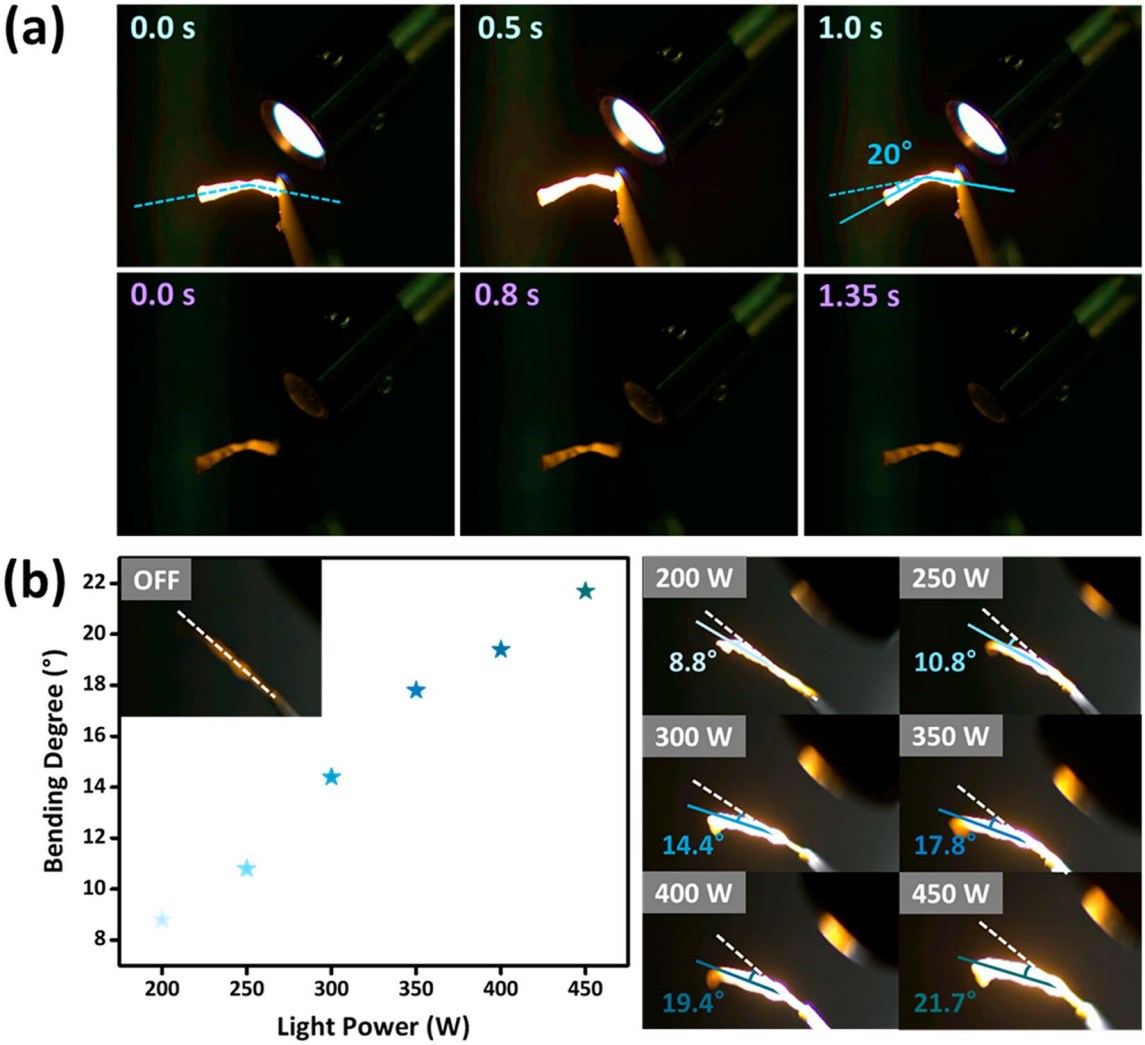
Photobending (PB) performance of MAPbBr_3_ QD/AzoPDMS composite film. (**a**) PB of MAPbBr_3_ QD/AzoPDMS composite film upon white light (stretched → bent) and 365 nm light (bent → stretched) irradiation. MAPbBr_3_ QD/AzoPDMS composite film showed 20° bending. (**b**) The bending degree of MAPbBr_3_ QD/AzoPDMS increased as the power of irradiated light increased.

**Figure 4:**
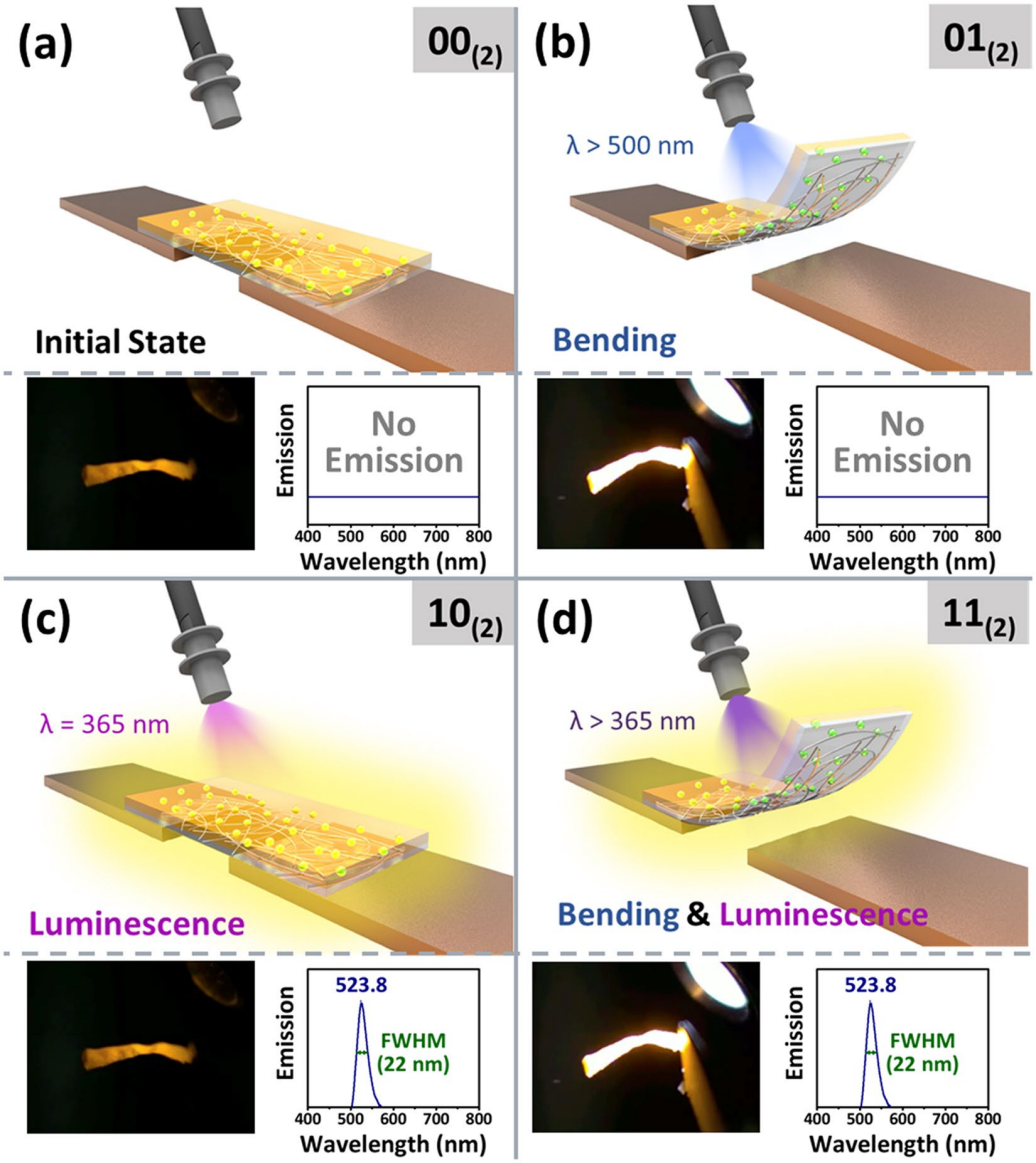
Independent multi-states of MAPbBr_3_ QD/AzoPDMS composite film. Four states induced by combination of PB and PL upon irradiation of different wavelengths of light on the MAPbBr_3_ QD/AzoPDMS nanocomposite. Diagrams indicate PB and PL for each state.

The three-dimensional structure of AzoPDMS changed upon irradiation via *cis*-to-*trans* isomerization of azobenzene. As a consequence, volume change of AzoPDMS resulted in the volume difference between the AzoPDMS layer and the silk fibroin layer inducing PB of the film upon irradiation^1^. As the film bent upon irradiation, it bent in a direction where AzoPDMS layer folds outside. White light (200 to 2000 nm) and 365 nm light (365 nm bandpass filter; bandwidth of 12 nm, more detail in Experimental Section) were irradiated for PB of the bilayer film with Xe arc lamp (power of 450 W). The isomerization of azobenzene molecules occurred both ways via white light, however, the *cis-* to *trans-* transition was faster (1.0 sec) than the opposite direction (1.35 sec), being the *trans-*form to be dominant^1^. In other words, the film bent ~20° within 1.0 sec when white light was irradiated (*trans*-form), whereas the film stretched within 1.35 sec when 365 nm light was applied (*cis*-form) (Fig. 3). To note, we excluded the light of 500 nm or less in wavelengths instead of white light to solely show PB without PL as shown in Fig. 4(b). Since the number of MAPbBr_3_ QDs increases in proportion to the thickness of film, the film was synthesized relatively thick to enhance the PL intensity; nanocomposite film consisted of 32.4 μm thick MAPbBr_3_ QD/AzoPDMS layer with 10 μm silk fibroin layer, while previous film consisted of 16 μm thick MAPbBr_3_ QD/AzoPDMS layer and the same thickness of silk fibroin layer^1^. Therefore, the bending angle of the film was less (~20°) than our previous work (~90°) which was much thinner. In addition, the bending degree of MAPbBr_3_ QD/AzoPDMS linearly increased as the intensity of irradiated light increased as shown in Fig. 3(b).

Our multi-switch can act as a four-states switch owing to independent changes in PL and structure of the film via (range of) wavelength(s) of irradiated light; the film bent when the wavelength of light greater than 500 nm was irradiated, whereas stretched upon irradiation of 365 nm light. Also, the composite film emitted light when wavelength of irradiated light was lower than 500 nm. By combining two phenomena, the MAPbBr_3_ QD/AzoPDMS composite film exhibited bending and/or luminescence depending on the wavelength of applied light. When 365 nm light was applied, the film emitted 523 nm light by PL. Under the light of wavelength higher than 365 nm, bending and light emission occurred simultaneously by isomerization of azobenzene and PL of MAPbBr_3_ QDs, respectively. Upon irradiation of light wavelength higher than 500 nm, only bending occurred because MAPbBr_3_ QDs did not absorb light. Since the two properties – PB and PL – of nanocomposite film are reversible and independent, we have indirectly confirmed that the switching of 01_(2)_ state into 10_(2)_ state are reversible; as the wavelength of irradiation λ converted from longer than 500 nm to 365 nm, stretching and luminescence occurred independently within ~1.35 second and almost instantly, respectively. Importantly, the switching between states was reversible which can possibly enable us to repetitively check the position and/or shape of the film by light emission. Furthermore, it can be applied as a logic gate if multiple states can be converted into electric signals or a real-time location sensor if observed with confocal microscope while the film induces stimulation via bending. We believe our composite film will play a pioneering role in materials with multiple independent states for various opto-electronic or biological applications.

## Conclusion

In this study, we have developed a multi-stimuli responsive material composed of photo-responsive polymer and perovskite QDs which exhibits both PB and PL properties. As-prepared multi-state switch was independently controlled by light of different wavelengths without any physical contact, which resulted in four states: (i) luminescence OFF and stretched (at initial state; [00_(2)_]), (ii) luminescence OFF and bent (at λ > 500 nm; [01_(2)_]), (iii) luminescence ON and stretched (at λ = 365 nm; [10_(2)_]), and (iv) luminescence ON and bent (at λ > 365 nm; [11_(2)_]). Multi-state material can be advantageous in mimicking complicated phenomena such as biological reactions, or can be applied as logic gates converting the information into electrical signals.

## Experimental

### Materials

Toluene (99.8%), methyl alcohol (99.9%), and nitric acid (68.0–70.0%) were purchased from Samchun. Tetrahydrofuran (THF) was obtained from Duksan. Hydrochloric acid (36.5–38.0%) was ordered from J.T.Baker. Silk fibroin (5% solution) was purchased from Advanced BioMatrix. Sodium nitrite, 6-bromo-1-hexene, chloroplatinic acid hexahydrate (H_2_PtCl_6_ ∙ 6H_2_O), decanoic acid, hydrazine monohydrate, silver acetate, dodecylamine (DDA), lead(II) bromide, methylammonium bromide (MABr), 4-methoxyaniline, sodium hydride, and tetrabutylammonium borohydride (TBAB) were obtained from Sigma-Aldrich. AZ-5214E was used as photoresist (PR). Sodium hydroxide, phenol, and petroleum ether were ordered from Daejung. N,N-dimethylformamide (DMF) was purchased from Honeywell. Silicone elastomer base and silicone elastomer curing agent (Sylgard 184) were obtained from Dow Corning.

### Instruments

Ultraviolet-visible (UV-Vis) absorption spectra were obtained by UV-Vis spectrometer (Lambda 365, Perkin-Elmer, Inc.), and scanning electron microscopy (SEM) images were taken by JSM-7100F (JEOL Ltd.). X-ray diffraction (XRD) spectra were obtained by X-ray diffractometer from SmartLab (Rigaku Corp.). Photoluminescence spectra were obtained by fluorescence spectrometer F-7100 (HITACHI, Ltd.). Xenon research arc lamp source (66924-450XV-R1, Newport Corp., USA) with an output power of 450 W was used for an irradiation, which was housed in a dark Faraday cage in order to minimize undesirable illuminance and electrostatic effects from external environment. Air motor was also installed to minimize thermal effects. A circular light source with a diameter of 50 mm was used, and the distance from light source to film was 60 mm. Light of different wavelengths have been applied by using 503 nm high pass filter, 365 nm to 503 nm band pass filter, 365 nm band pass filter (MaxLamp Hg01-365-25, Semrock, USA) and 365 nm low pass filter. The transmission band of 365 nm band pass filter ranged from 360 to 372 nm with more than 93% of transmission, and the center wavelength was 365 nm.

## Methods

### Synthesis of AzoPDMS

1-(4-(Hex-5-enyloxy)phenyl)-2-phenyldiazene incorporated poly(dimethylsiloxane) (AzoPDMS) was synthesized by previously reported method^1^. In AzoPDMS synthesis, Sylgard 184 silicone elastomer kit (Dow Corning) was used as prepolymer and chloroplatinic acid hexahydrate (H_2_PtCl_6_ ∙ 6H_2_O) was used as a catalyst for polymerization. An analytical grade of toluene (Samchun, 99.5%) was used as a solvent.

### Synthesis of AgNP/AzoPDMS and Silk Fibroin Bilayer Film

AgNP/AzoPDMS and silk fibroin bilayer film was fabricated in order to prepare porous template for MAPbBr_3_ QD growth. To note, AgNPs were used in this work since AzoPDMS was fragile in strong acidic condition (e.g. in our previous work, aqua regia was used for etching AuNPs)^35^, and AgNPs were synthesized according to the method described by Klajn and co-workers^36^. First, AZ-5214E, a photoresist, was spin-coated onto the wafer for 30 seconds at 3000 rpm followed by curing for 2 minutes at 120 °C. Afterwards, silk fibroin solution was drop-casted onto the PR layer for facile removal from the wafer and dried for 12 hours. 2 mL of AgNPs solution (NP concentration of 5.7 × 10^15^ mL^−1^) was added in 1 g AzoPDMS prepolymer with vigorous stirring. Then, the mixture of AgNP/AzoPDMS was spin-coated onto silk fibroin layer for 30 seconds by 1500 rpm. After curing, PR was removed with acetone in order to obtain AgNP/AzoPDMS and silk fibroin bilayer film.

### Synthesis of MAPbBr3 QD/AzoPDMS and Silk Fibroin Bilayer Film

In order to etch away AgNPs in AgNP/AzoPDMS and silk fibroin bilayer film, the film was alternately immersed between THF and 3 M HNO_3_ solution for 3 times. Each process lasted 15 minutes, and the film was washed with THF. The etched porous AzoPDMS and silk fibroin bilayer film was swollen in THF for 15 min and then immersed in methylammonium lead halide perovskite precursor solution (1.0 M of MAPbBr_3_, in anhydrous DMF) for 10 h. To make sure MAPbBr_3_ QDs to fully grow within in the pores, we repeated this process several times. Afterwards, the film was dried in vacuum oven at 70 °C for overnight.
